# Adenosquamous Carcinoma of the Cervix: A Population-Based Analysis

**DOI:** 10.3389/fonc.2021.652850

**Published:** 2021-07-22

**Authors:** Pengfei Cui, Xiaofeng Cong, Chen Chen, Lei Yang, Ziling Liu

**Affiliations:** Cancer Center, The First Hospital of Jilin University, Changchun, China

**Keywords:** adenosquamous carcinoma of the cervix, incidence, prognostic factors, nomogram, risk classification system

## Abstract

**Background:**

Due to the rarity of adenosquamous carcinoma of the cervix (ASCC), studies on the incidence, prognostic factors, and treatment outcomes of ASCC remain scarce. Therefore, we performed a retrospective population-based study to systematically investigate the characteristics of ASCC patients.

**Methods:**

Patients with a histopathologically confirmed diagnosis of ASCC were enrolled from the Surveillance, Epidemiology, and End Results database between 1975 and 2016. Univariate and multivariate Cox regression analyses were performed to identify the potential predictors of cancer-specific survival (CSS) in patients with ASCC. Selected variables were integrated to establish a predictive nomogram and the predictive performance of the nomogram was estimated using Harrell’s concordance index (C-index), calibration curve, and decision curve analysis (DCA).

**Results:**

A total of 1142 ASCC patients were identified and included in this study and were further randomized into the training and validation cohorts in a 7:3 ratio. The age-adjusted incidence of ASCC declined from 0.19 to 0.09 cases per 100,000 person-years between 2000 and 2017, with an annual percentage change of -4.05% (P<0.05). We identified age, tumor grade, FIGO stage, tumor size, and surgical procedure as independent predictors for CSS in ASCC patients and constructed a nomogram to predict the 3- and 5-year CSS using these prognostic factors. The calibration curve indicated an outstanding consistency between the nomogram prediction and actual observation in both the training and testing cohorts. The C-index was 0.7916 (95% CI: 0.7990-0.8042) and 0.8148 (95% CI: 0.7954-0.8342) for the training and testing cohorts, respectively, indicating an excellent discrimination ability of the nomogram. The DCA showed that the nomogram exhibited more clinical benefits than the FIGO staging system.

**Conclusions:**

We established and validated an accurate predictive nomogram for ASCC patients based on several clinical characteristics. This model might serve as a useful tool for clinicians to estimate the prognosis of ASCC patients.

## Introduction

Cervical cancer was the fourth most frequent malignancy and the leading cause of gynecologic cancer-associated mortality worldwide in 2018 (1). Although the introduction of the human papillomavirus (HPV) vaccine and effective cervical cancer screening strategies have resulted in a significant decline in the incidence and morbidity of cervical cancer, it continues to be the most prevalent gynecological malignancy in 28 countries and the leading cause of cancer-related deaths in 42 countries (1, 2). High incidence and mortality from cervical cancer persist in low- and middle-income countries, such as southern Africa, and are primarily attributed to the lack of effective prevention and high-quality screening strategies (1, 3). Histologically, squamous cell carcinoma of the cervix (SCCC) represents the most common subtype of cervical cancer, accounting for approximately 75% of all cervical cancers, followed by adenocarcinoma of the cervix (ADCC) with a proportion of approximately 15% (4). Unusual histological subtypes such as adenosquamous carcinoma and neuroendocrine tumors are rare. Adenosquamous carcinoma of the cervix (ASCC) is an infrequent malignant epithelial neoplasm characterized by the presence of both squamous cell and glandular differentiation according to the World Health Organization (WHO) classification of tumors of female reproductive organs (5).

Few reports have documented the survival outcome and prognostic factors in patients with ASCC; however, inconsistencies exist among these studies. Furthermore, most of these studies considered ASCC a type of ADCC; thus, specific studies on patients with ASCC are lacking. Besides, there is a paucity of published literature related to the epidemiology, clinical characteristics, and response to therapy against ASCC. Therefore, we conducted a retrospective study based on a large population to systematically investigate the characteristics of patients with ASCC.

## Materials and Methods

### Data Source

Data including the incidence, demographic information, clinical characteristics, treatment modalities, and survival information were collected from the Surveillance, Epidemiology, and End Results (SEER) database of the National Cancer Institute using SEER*Stat software version 8.3.8. The SEER database is universally considered one of the most authoritative and reliable programs for oncologists performing cancer research because of its large sample size, regular updates, and high-quality data (6).

### Study Population

Women diagnosed with cervical cancer between 1975 and 2016 were preliminarily identified from the SEER database. Tumor site and histology were coded according to criteria specified by the WHO International Classification of Diseases for Oncology. Eligible patients with a histopathologically confirmed diagnosis of cervical cancer were included, and patients with more than one primary tumor were excluded from this study. The detailed inclusion and exclusion criteria in the present study are shown in **Figure S1**. In this study, demographic data included age at diagnosis (≤60 or >60 years), marital status (married, unmarried, or widowed), and race (black, white, or other). Clinical and histopathological characteristics included the grade (I/II: well/moderately differentiated, III/IV: poorly differentiated/undifferentiated or unknown), the International Federation of Gynecology and Obstetrics (FIGO) stage (I, II, III, or IV), and tumor size (≤3.5 cm, >3.5 cm, or unknown). The treatment modalities included surgery (no or yes), radiotherapy (no/unknown or yes), and chemotherapy (no/unknown or yes). Surgery was further classified into the following types to investigate the influence of different surgical approaches on the survival of patients with ASCC: none performed, local tumor destruction (LTD), total hysterectomy without the removal of tubes and ovaries (THR-RTO), total hysterectomy with the removal of tubes and ovaries (THR+RTO), and radical hysterectomy (RHR). Survival data included the survival length in months, and vital status (cause-specific death, death for other cause, or alive). Data on the FIGO stage was not available in the SEER database; thus, in our study, it was defined according to the TNM staging system.

Cancer-specific survival (CSS) was considered the primary endpoint in this study and was defined as the survival time from the time of diagnosis to the time of death from ASCC or last follow-up.

### Statistical Analysis

Categorical data are presented as frequency and proportion. Chi-square test was used to analyze the differences between categorical variables among different groups. The Kaplan-Meier method was used to construct survival curves, and the log-rank test was used to compare the CSS rates in patients with different histological subtypes, different treatment modalities, and different risk groups. ASCC patients were randomized into training and validation cohorts in a 7:3 ratio. The Cox proportional hazard model was used to identify the independent prognostic factors of CSS in the training cohort. Statistically significant risk factors were integrated to establish the predictive nomograms of the 3-year and 5-year CSS rates. Using Harrell’s concordance index (C-index), the discrimination and calibration of nomograms were measured to evaluate the predicted probabilities of the nomogram in both the training and validation cohorts. Calibration (1000 bootstrap resamples) curves were plotted to compare the consistency between the nomogram prediction and actual survival probability at 3- and 5-year CSS. The net reclassification improvement (NRI) and integrated discrimination improvement (IDI) were calculated to assess the difference in predictive power between the constructed nomogram and the traditional FIGO stage model. Decision curve analysis (DCA) was used to evaluate the clinical benefits of the nomogram model and the FIGO staging system. A two-sided P-value of <0.05 was considered statistically significant.

### Statistical Software Programs

The age-adjusted incidence (from 2000 to 2017) of different histological subtypes was calculated using the SEER*Stat software. Other statistical calculations including the survival curve, calibration plot, DCA, C-index, NRI, and IDI values were performed using R software program version 4.0.2. The incidence-time curve was plotted using Microsoft Excel version 2019. The cut-off point of the tumor size and nomogram scores for the risk stratification was determined using X-tile version 3.6.1. Relevant R packages included “rms”, “survival”, “compare”, “nricens”, “regplot”, “mstate”, “survminer”, “foreign”, “caret”, “lattice”, and “nomogramEX” (https://cran.r-project.org/).

## Results

### Incidence, Proportion, Clinicopathological Characteristics, and Treatment Outcome in Cervical Cancer in Women With Different Histological Subtypes

**Figure 1:**
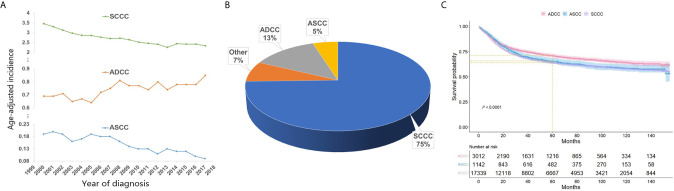
The age-adjusted incidence of ASCC, ADCC, and SCCC between 2000 and 2017 **(A)**; Proportion of different histological subtypes of cervical cancer **(B)**; Kaplan-Meier curves showing cancer-specific survival of patients with ASCC, ADCC, and SCCC **(C)**.

Overall, the age-adjusted incidence of ASCC and SCCC has significantly decreased from 2000 to 2017 in the US, whereas that of ADCC has gradually increased. Indeed, the incidence of ASCC declined from 0.19 to 0.09 cases per 100,000 person-years between 2000 and 2017, with an annual percentage change (APC) of -4.05% (P<0.05) (**Figure 1A**). The incidence of SCCC declined from 3.46 to 2.34 cases per 100,000 person-years, and the APC was -2.38% (P<0.05) (**Figure 1A**). However, the incidence of ADCC slowly increased from 0.69 to 0.85 cases per 100,000 person-years during the observation period, with an APC of +1.16% (P<0.05) (**Figure 1A**).

A total of 23,215 eligible cervical cancer patients meeting the selection criteria were identified and included in the present study. Among them, 17,339 (75%) patients had SCCC, 3,012 (13%) had ADCC, 1,142 (5%) had ASCC, and 1,722 (7%) patients presented with other histological subtypes (**Figure 1B**).

Kaplan-Meier analysis revealed that patients with ASCC and SCCC exhibited comparable CSS (P=0.336), and patients with ADCC exhibited a better treatment outcome compared with those with ASCC and SCCC (P=0.007 and P<0.001, respectively). The 5-year CSS rate was 74% for ADCC, 69% for ASCC, and 68% for SCCC (**Figure 1C**).

The demographic profiles of patients with ASCC indicated that patients were younger, mostly married; presented with higher tumor grades, earlier FIGO stage, and smaller tumor size; and more patients underwent radical surgery than those with SCCC. However, the proportion of individuals who were young, unmarried, Black, had poor tumor differentiation, advanced stage, larger tumor size, and receiving radiotherapy or chemotherapy among ASCC patients was higher than that in ADCC patients. The demographic and clinical characteristics of cervical cancer patients with different histological subtypes are summarized in **Table S1**.

### Clinicopathological Characteristics of Patients With ASCC

A total of 1,142 ASCC patients were further analyzed. The demographic characteristics of ASCC patients revealed that most women were younger (84.2%) and married (62.7%). In addition, most of the patients were White (75.7%). The clinical characteristics of cancer suggested that more than half of the ASCC patients presented with grade III/IV (51.5%) tumors, and nearly half of all patients presented with FIGO stage I (49.5%). The number of patients with a tumor size ≤3.5 cm (38.0%) was comparable to those with a tumor size >3.5 cm (36.7%). Although the proportion of ASCC patients who underwent radical surgery, including THR+RTO (22.3%) and RHR (32.9%) was high, a significant proportion of patients received radiotherapy (60.5%) and chemotherapy (52.6%). Clinicopathological characteristics of ASCC patients are presented in **Table 1**.

**Table 1:** Clinical features of ASCC patients in the total, training, and validation cohorts.

Variables	Total population	Training cohort	Validation cohort	*P* value
	N=1142	N=802	N=340	
**Age (years)**				0.242
≤60	962 (84.2)	669 (83.4)	293 (86.2)	
>60	180 (15.8)	133 (16.6)	47 (13.8)	
**Marital status**				0.392
Married	716 (62.7)	497 (62.0)	219 (64.4)	
Unmarried	352 (30.8)	248 (30.9)	104 (30.6)	
Widowed	74 (6.5)	57 (7.1)	17 (5.0)	
**Race**				0.169
Black	132 (11.6)	102 (12.7)	30 (8.8)	
White	865 (75.7)	599 (74.7)	266 (78.2)	
Other^*^	145 (12.7)	101 (12.6)	44 (12.9)	
**Grade** ^#^				0.550
I/II	328 (28.7)	223 (27.8)	105 (30.9)	
III/IV	588 (51.5)	420 (52.4)	168 (49.4)	
Unknow	226 (19.8)	159 (19.8)	67 (19.7)	
**FIGO stage**				0.226
I	565 (49.5)	386 (48.1)	179 (52.6)	
II	161 (14.1)	120 (15.0)	41 (12.1)	
III	260 (22.8)	191 (23.8)	69 (20.3)	
IV	156 (13.7)	105 (13.1)	51 (15.0)	
**Tumor size (cm)**				0.897
≤3.5	434 (38.0)	304 (37.9)	130 (38.2)	
>3.5	419 (36.7)	292 (36.4)	127 (37.4)	
Unknow	289 (25.3)	206 (25.7)	83 (24.4)	
**Surgical procedure**				0.911
None	348 (30.5)	242 (30.2)	106 (31.2)	
LTD	106 (9.3)	71 (8.9)	35 (10.3)	
THR-RTO	57 (5.0)	41 (5.1)	16 (4.7)	
THR+RTO	255 (22.3)	183 (22.8)	72 (21.2)	
RHR	376 (32.9)	265 (33.0)	111 (32.6)	
**Radiotherapy**				0.622
No	451 (39.5)	313 (39.0)	138 (40.6)	
Yes	691 (60.5)	489 (61.0)	202 (59.4)	
**Chemotherapy**				0.691
No	541 (47.4)	383 (47.8)	158 (46.5)	
Yes	601 (52.6)	419 (52.2)	182 (53.5)	

^*^including Asian, American Indian and Alaska Native.

^#^I, well differentiated; II, moderately differentiated; III, poorly differentiated; IV, undifferentiated.

FIGO, the International Federation of Gynecology and Obstetrics; LTD, local tumor destruction; THR-RTO, total hysterectomy without removal of tubes and ovaries; THR+RTO, total hysterectomy with removal of tubes and ovaries; RHR, radical hysterectomy.

### Independent Predictors for CSS

ASCC patients were randomized into the training and validation cohorts in a 7:3 ratio, and no significant difference was observed between the two cohorts for each of the variables (**Table 1**). The independent risk factors for CSS were screened in the training cohort. Univariate analyses revealed that age, marital status, tumor grade, FIGO stage, tumor size, surgical procedure, radiotherapy, and chemotherapy were significant prognostic factors for the CSS of patients with ASCC (all P <0.05) (**Table 2**). However, race did not significantly influence CSS (**Table 2**). A subsequent multivariate Cox regression analysis indicated that age, grade, FIGO stage, tumor size, and surgical procedure were independent predictors of CSS of patients with ASCC (all P <0.05) (**Table 2**).

**Table 2:** Univariate and multivariate Cox analyses for CSS of ASCC patients in the training cohort.

Variables	Univariate analysis			Multivariate analysis		
	HR	95% CI	*P* value	HR	95% CI	*P* value
**Age (years)**						
>60 *vs* ≤60	2.088	1.587-2.748	<0.001	0.668	0.481-0.929	0.016
**Marital status**						
Unmarried *vs* Married	1.002	0.767-1.311	0.986	1.027	0.781-1.352	0.848
Widowed *vs* Married	2.118	1.442-3.110	<0.001	1.148	0.735-1.793	0.544
**Race**						
Black *vs* White	0.759	0.508-1.134	0.179			
Other^*^ *vs* White	1.325	0.956-1.837	0.091			
**Grade** ^#^						
III/IV *vs* I/II	1.783	1.312-2.423	<0.001	1.427	1.047-1.946	0.025
Unknow *vs* I/II	1.688	1.171-2.434	0.005	1.318	0.902-1.926	0.154
**FIGO stage**						
II *vs* I	2.917	1.992-4.270	<0.001	1.697	1.082-2.662	0.021
III *vs* I	3.479	2.505-4.832	<0.001	3.052	2.063-4.514	<0.001
IV *vs* I	10.497	7.523-16.646	<0.001	5.594	3.688-8.484	<0.001
**Tumor size (cm)**						
>3.5 *vs* ≤3.5	4.142	2.934-5.848	<0.001	2.090	1.428-3.061	<0.001
Unknow *vs* ≤3.5	4.176	2.921-5.973	<0.001	2.231	1.511-3.294	<0.001
**Surgical procedure**						
LTD *vs* None	0.343	0.219-0.536	<0.001	0.624	0.390-0.998	0.049
THR-RTO *vs* None	0.235	0.120-0.461	<0.001	0.645	0.312-1.335	0.237
THR+RTO *vs* None	0.263	0.189-0.366	<0.001	0.454	0.314-0.657	<0.001
RHR *vs* None	0.170	0.122-0.237	<0.001	0.358	0.243-0.528	<0.001
**Radiotherapy**						
Yes *vs* No	2.570	1.933-3.418	<0.001	1.029	0.704-1.506	0.882
**Chemotherapy**						
Yes *vs* No	2.526	1.950-3.271	<0.001	0.763	0.537-1.084	0.131

^*^including Asian, American Indian and Alaska Native.

^#^I, well differentiated; II, moderately differentiated; III, poorly differentiated; IV, undifferentiated.

HR, Hazard ratio; 95% CI ,95% confidence intervals; FIGO, the International Federation of Gynecology and Obstetrics; LTD, local tumor destruction; THR-RTO, total hysterectomy without removal of tubes and ovaries; THR+RTO, total hysterectomy with removal of tubes and ovaries; RHR, radical hysterectomy.

To evaluate the relationship between radiotherapy or chemotherapy and CSS in ASCC patients, the patients were divided into three groups including localized (stage I), regional (stage II-III), and distant (stage IV) groups. Kaplan-Meier analysis revealed that the CSS rate in patients receiving radiotherapy or chemotherapy was significantly lower than that in patients who did not receive radiotherapy (5-year CSS rate: 91% *vs*. 71%, P<0.001) or without chemotherapy (5-year CSS rate: 87% *vs*. 73%, P<0.001) in the localized group (**Figure S2**).

### Nomogram for the Prediction of CSS

**Figure 2:**
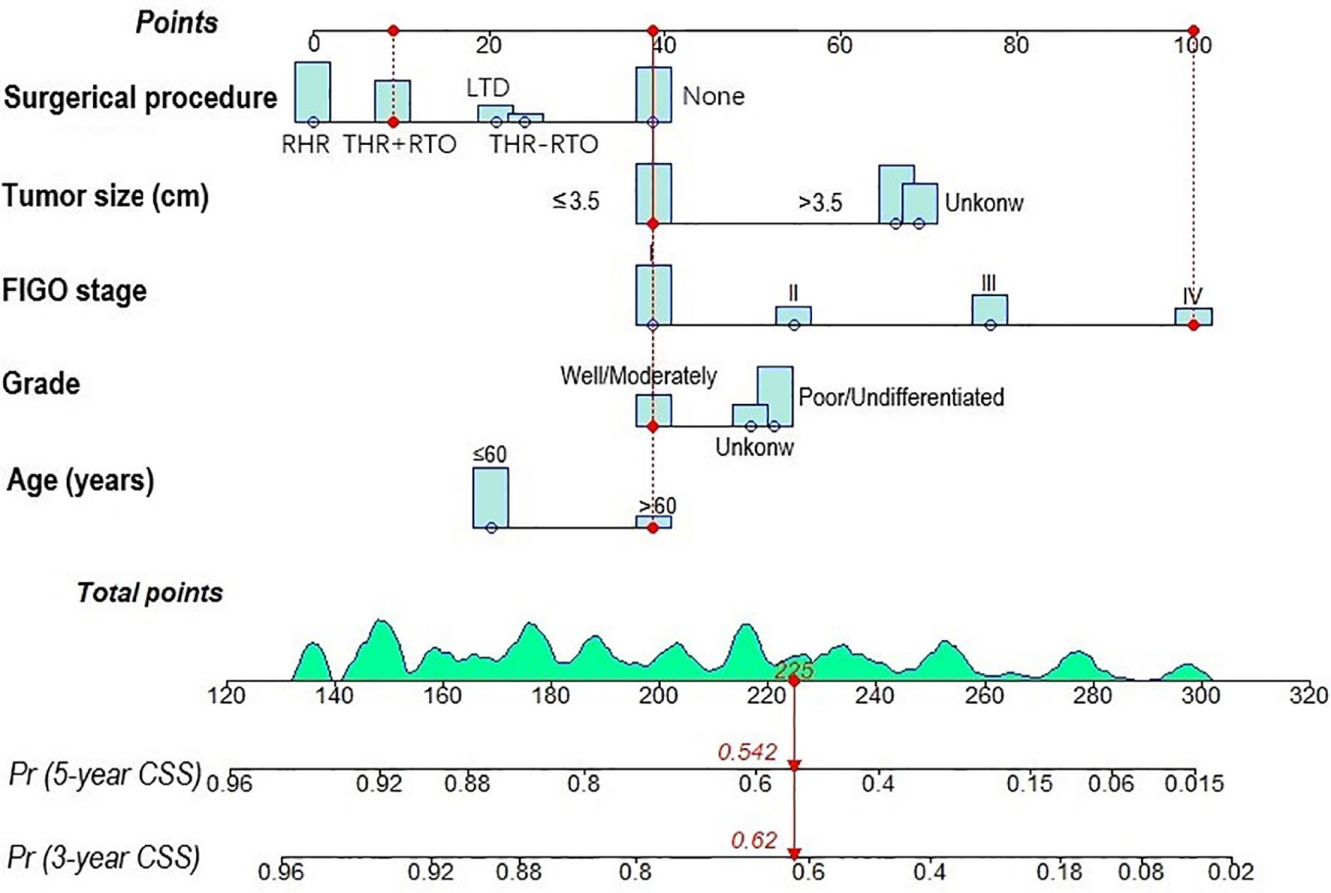
Nomogram for predicting 3- and 5-year cancer-specific survival of ASCC patients.

A predictive nomogram model was constructed according to the independent risk factors for CSS to predict the 3- and 5-year CSS (**Figure 2**). The nomogram findings indicated that the FIGO stage had the widest score span (61 scores), suggesting that FIGO stage played a pivotal role in predicting the prognosis of ASCC patients among all variables, followed by surgical procedure (39 scores), tumor size (30 scores), age (19 scores), and tumor grade (13 scores). The scores of different levels of each variable are presented in **Table 3**.

**Table 3:** Scores of each variable in the nomogram.

Variables	Level	Scores	Variables	Level	Scores
**Age (years)**	≤60	20	**Tumor size**	≤3.5	39
	>60	39	**(cm)**	>3.5	66
**Grade** ^#^	I/II	39		Unknow	69
	III/IV	52	**Surgical procedure**	None	39
	Unknow	50		LTD	21
**FIGO stage**	I	39		THR-RTO	24
	II	55		THR+RTO	9
	III	77		RHR	0
	IV	100			

^#^I, well differentiated; II, moderately differentiated; III, poorly differentiated; IV, undifferentiated.

FIGO, the International Federation of Gynecology and Obstetrics; LTD, local tumor destruction; THR-RTO, total hysterectomy without removal of tubes and ovaries; THR+RTO, total hysterectomy with removal of tubes and ovaries; RHR, radical hysterectomy.

**Figure 3:**
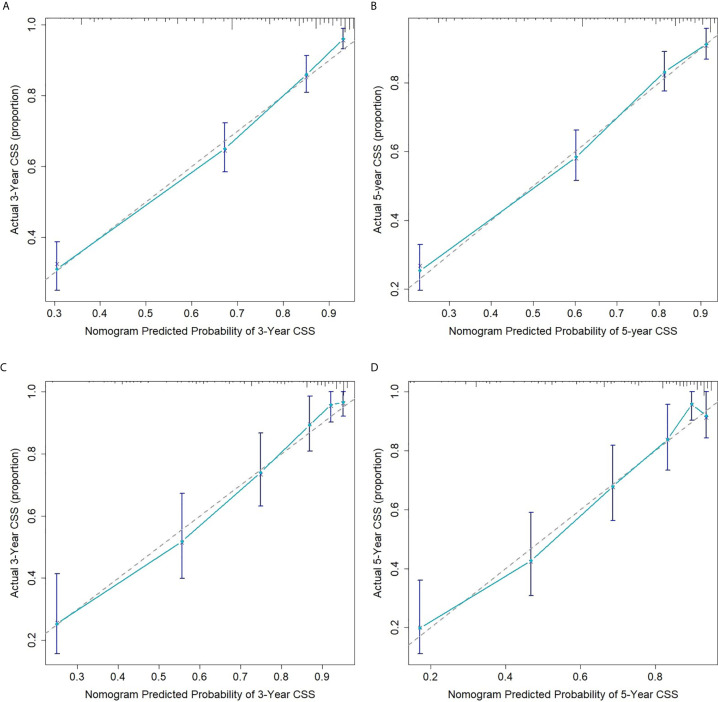
Calibration curves for 3- **(A)** and 5-year **(B)** CSS of ASCC patients in the training cohort; Calibration curves for 3- **(C)** and 5-year **(D)** CSS of ASCC patients in the validation cohort.

The internal and external validation of nomograms was performed in the training and validation cohorts, respectively. The C-index was calculated in the training cohort (internal validation) and testing cohort (external validation) with a value of 0.7916 (95% CI: 0.7990-0.8042) and 0.8148 (95% CI: 0.7954-0.8342), respectively, suggesting a strong discrimination power of the predictive model. The calibration curves revealed a satisfactory correlation between the prediction of the nomogram and the actual observation in both the training and validation cohorts, demonstrating the excellent calibration ability of the nomogram model (**Figure 3**).

### Comparison of the Nomogram Model With the FIGO Staging System

Comparing the predictive performance between the novel nomogram model and classical FIGO staging system was achieved by analyzing the NRI values, IDI values, change in C-indexes, and DCA.

The NRI value in the training cohort was 0.6278 (95% CI: 0.4248-0.8355) for 3 years of follow-up and 0.5992 (95% CI: 0.4142-0.7862) for 5 years of follow-up; the corresponding values in the validation cohort were 0.2718 (95% CI: 0.1348-0.7513) and 0.3053 (95% CI: 0.1659-0.7216), respectively. These results indicated that the new model exhibited improved predictive performance compared with the conventional FIGO staging system. Similarly, the IDI values for the 3- and 5-year follow-up were 0.0865 and 0.0916 (all P<0.001), respectively, and 0.0438 and 0.0506, respectively (all P<0.001), in the validation group. The change in C-index was 0.0628 (95% 0.0609-0.0647) in the training cohort and 0.0454 (95% 0.0437-0.0471) in the validation cohort. These values suggested that the constructed nomogram exhibited superior predictive performance compared with the FIGO staging system. All results are presented in **Table 4**.

**Table 4:** Value of the NRI, IDI, and C-indexes of the nomogram and FIGO staging system in both the training and validation cohorts.

Values	Training cohort		*P* value	Validation cohort		*P* value
**NRI**						
3-year CSS	0.6278	0.4248-0.8355	--	0.2718	0.1348-0.7513	--
5-year CSS	0.5992	0.4142-0.7862	--	0.3053	0.1659-0.7216	--
**IDI**						
3-year CSS	0.0865	0.0843-0.0887	<0.001	0.0438	0.0425-0.0451	<0.001
5-year CSS	0.0916	0.0901-0.0931	<0.001	0.0506	0.0490-0.0522	<0.001
**C-indexes**						
Nomogram	0.7916	0.7990-0.8042	--	0.8148	0.7954-0.8342	--
FIGO stage	0.7288	0.7143-0.7433	--	0.7694	0.7483-0.7905	--
Change	0.0628	0.0609-0.0647	<0.001	0.0454	0.0437-0.0471	<0.001

CSS, cancer-specific survival; NRI, net reclassification improvement; IDI, integrated discrimination improvement; C-indexes, concordance indexes; FIGO, the International Federation of Gynecology and Obstetrics.

**Figure 4:**
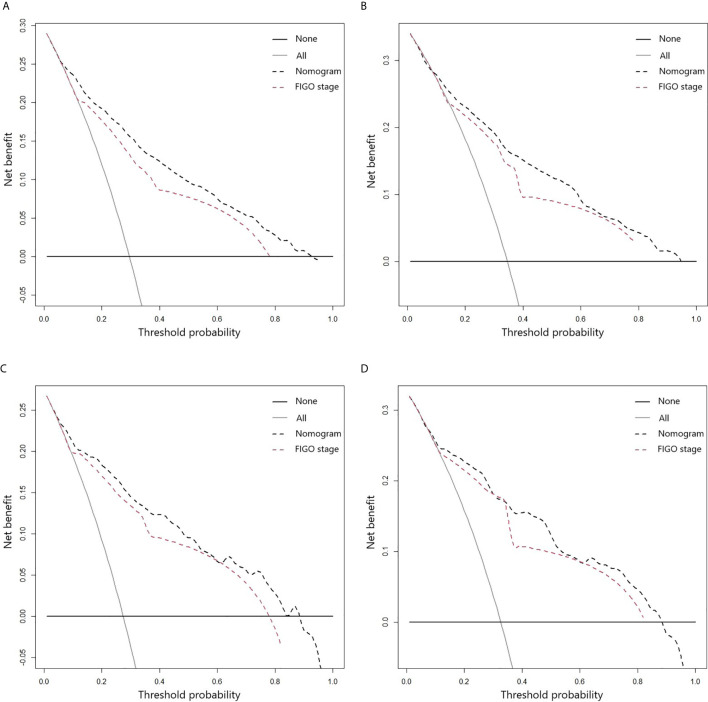
DCA curves for 3- **(A)** and 5-year **(B)** CSS of ASCC patients in the training cohort; DCA curves for 3- **(C)** and 5-year **(D)** CSS of ASCC patients in the validation cohort.

The clinical benefits of the nomogram and the FIGO stage were also analyzed, and the results are presented using DCA models (**Figure 4**). The DCA models revealed that the proposed nomogram was superior to the FIGO staging system in evaluating clinical benefits, indicating more reliable predictions using the nomogram model.

### The Novel Risk-Stratification System

**Figure 5:**
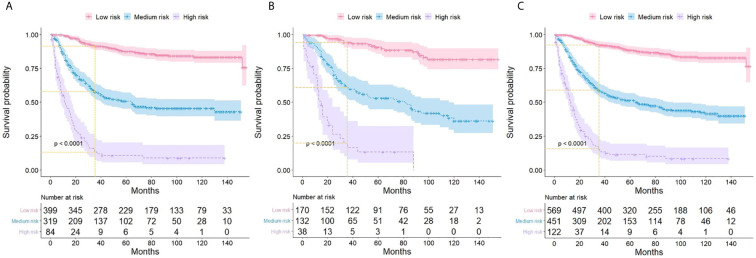
Kaplan-Meier curves showing the cancer-specific survival of ASCC patients with different risk groups in training **(A)**, validation **(B)**, and the total cohort **(C)**.

Based on the corresponding nomogram score of each variable, the total score was calculated for all ASCC patients. The median total score was 201, and ranged from 137 to 299 in the training group. Patients were divided into low- (≤200), medium- (201-260), and high-risk (>260) groups according to these risk scores. Kaplan-Meier analysis showed marked differences in the CSS of these three risk groups, and the 3-year CSS rate was found to be 91% for the low-risk group, 58% for the medium-risk group, and 13% for the high-risk group (**Figure 5**). These results suggested that the novel risk-stratification system exhibited a strong ability to identify high-risk ASCC patients, which was further tested in the validation and total cohorts (**Figure 5**).

## Discussion

Since ASCC is a rare cervical malignancy, only limited reports are available regarding its clinicopathological characteristics and treatment outcomes. Furthermore, the majority of these reports include retrospective analyses on a very small sample size and meta-analysis that show remarkable inconsistencies in results (7–10). To the best of our knowledge, the present study is the first and the most extensive population-based study to assess the incidence, prognostic factors, and treatment outcomes of ASCC patients. In particular, we also developed a novel prognostic nomogram model to predict the 3- and 5-year CSS in ASCC patients in this study.

According to the reported data (11), the present study also indicated that the age-adjusted incidence rate of SCCC gradually decreased, while the ADCC incidence slightly increased between 2000 and 2017. The APC was -2.38% and 1.16% for SCCC and ADCC, respectively (all P<0.05). The incidence of ASCC remained low, with a range of 0.09-2.0 cases per 100,000 person-years relative to SCCC and ADCC. Moreover, the incidence of ASCC significantly decreased with an APC of -4.05% from 2000 to 2017 (P<0.05). Consistent with previous findings (12, 13), the present study further confirmed that ASCC was more poorly differentiated and characterized by smaller tumor size. The present study also revealed significant differences in age, marital status, FIGO stage, and surgical procedure between ASCC and SCCC patients.

The difference in survival outcomes among SCCC, ASCC, and ADCC patients remains unexplained. A previous study comprising 161 cases indicated that the survival rate in ASCC patients was significantly lower than that in SCCC patients (5-year overall survival rate: 75% *vs*. 52%, P=0.006), which was attributed to differences in FIGO stage, pelvic lymph node involvement, and vascular invasion (7). Studies have reported that ADCC and ASCC had a similar survival outcome and were significantly poorer than that of SCCC patients in the early stage of the tumor (14–16). However, some studies have reported that the ASCC subtype is a poor predictor for survival in patients with an advanced tumor stage (12). Our results also revealed that women with ASCC and SCCC had a comparable CSS rate and poorer prognosis than ADCC patients, without considering the clinical stage. The proportion of stage I patients in the ADCC group (60%) was higher than that in the other two groups (49.5% for ASCC and 43.5% for SCCC), which resulted in better survival. The inconsistent results among different studies are mainly caused by the difference in exclusion and inclusion criteria. Notably, our study was conducted on a much larger sample size than previous studies and comprised 1,142 ASCC patients.

The present study is the first to perform a comprehensive analysis of the prognostic factors and treatment responses among ASCC patients. Univariate and multivariate COX regression models revealed that age, tumor grade, tumor size, FIGO stage, and surgical methods were independent risk factors. A nomogram was constructed to evaluate the 3- and 5-year CSS in ASCC patients. The predictive model indicated that FIGO stage was the most vital predictive factor. A single-center study also demonstrated that FIGO stage was the most potent prognostic factor in ADCC, and that the 5-year overall survival rate for patients with stage I reached 92%, in contrast to only 40% for those with stage II-IV ASCC (17).

Several reports regarding cervical carcinoma focus on certain risk factors for survival outcomes. Ample evidence indicates that tumor differentiation and clinical stage greatly affect the survival of patients with cervical cancer, and poor differentiation and high stage contribute to worse survival (18). Surgery is considered an essential initial treatment for prolonging the survival of patients with cervical tumors. The present study also demonstrated that invasive surgical methods such as RHR could significantly reduce the death hazard compared to non-surgery patients (HR: 0.358, 95% CI: 0.243-0.528, P<0.001). Previous reports have also indicated that the incidence of ovarian metastasis in non-squamous histological subtypes increases compared to SCCC; hence, bilateral oophorectomy is actively recommended in patients with ADCC (19, 20). In contrast, a large population-based study has reported that survival is not significantly prolonged by radical surgery in the early stage of microinvasive adenocarcinoma, suggesting the unsuitability of the method for these patients (21). In our study, the nomogram score in THR+RTO was 9, which was lower than 28 for THR-RTO, suggesting that RTO presented a survival advantage. It is noteworthy that there was no direct comparison between THR+RTO and THR-RTO in this study. In terms of tumor size, the conclusions of previous studies were relatively consistent with ours (22–24). Notably, irrespective of the FIGO stage and histological subtype, tumor size exhibits a significant impact on the survival of patients with cervical cancer, and larger primary tumors significantly shorten the survival compared to smaller ones (25–27). The results from our study were in agreement with those from published studies. The mortality risk was significantly higher in ASCC patients with large tumor sizes (more than 3.5 cm) according to both univariate and multivariate models. We found that age played a key role in the nomogram model. Older age of more than 60 years was identified as a high-risk factor for CSS in ASCC patients. An early study involving more than 10,000 cervical cancer cases revealed that the 5-year survival rate was 69% in patients < 40 years, and 45% for those ≥ 40 years regardless of stage. Moreover, the investigators also found that the 5-year survival rate gradually decreases with an increase in age (28). In contrast, another recent report indicated that younger age represented a detrimental factor in the early stage of cervical carcinoma, which may be attributed to a high percentage of young women with invasive diseases (29).

Surprisingly, radiotherapy and chemotherapy were not identified as independent prognostic factors for CSS in ASCC patients by multivariate cox analysis in this study. Indeed, the univariate cox analysis revealed that both radiotherapy and chemotherapy significantly increased the mortality hazard (HR=2.570; 95% CI: 1.933-3.418; P<0.001 and HR=2.526; 95% CI: 1.950-3.271; P<0.001, respectively). Therefore, patients from the training group were divided into three subgroups according to different FIGO stages to assess the efficacy of radiotherapy and chemotherapy on survival. Kaplan-Meier survival analysis indicated that adjuvant radiotherapy or chemotherapy after surgery significantly shortened the CSS of patients in the local stage subgroup. Chemotherapy significantly improved CSS only in the distant stage subgroup. Radiotherapy slightly improved CSS in the regional and distant stage subgroup, but the result was not statistically significant. Overall, our data revealed that adjuvant chemotherapy or radiotherapy was not strongly recommended in patients with ASCC at an early stage. Currently, there is a consistent opinion that chemotherapy alone following surgery is not recommended for patients at the early stage of cervical cancer, and radiotherapy or concurrent chemoradiation therapy can reduce recurrence and improve survival, and is instead recommended as the standard adjuvant therapy in patients with risk factors (30, 31). Since the SEER database did not have data regarding stromal invasion, lymphovascular space invasion, and conditions of margin and parametrial involvement, further stratification of patients according to these risk factors could not be performed. Furthermore, data concerning the administration and sequence of radiotherapy and chemotherapy were not available in the database; hence, the specific therapeutic patterns remain unknown. Therefore, we could not compare different treatment strategies among different risk groups.

The traditional FIGO stage is closely associated with the prognosis of cervical carcinoma and used as the primary reference by clinicians to make the clinical diagnosis. However, this staging system is predominantly based on tumor characteristics, including tumor size, depth of invasion, lymph node involvement, and distant organ metastasis. Other parameters such as age, marital status, tumor grade, and treatment choice are not considered in the FIGO staging system (32). Therefore, in this study, we integrated demographic and clinicopathological data, developed a novel prognostic model, and successfully established a predictive nomogram model for patients with ASCC. All variables integrated into this predictive model were prevalent and easily accessible while treating patients. Based on the corresponding score of each variable, the CSS probability of ASCC patients was intuitively predicted by the nomogram model. Besides, both internal and external validation based on calibration curves, C-indexes, and DCA curves demonstrated that this prognostic nomogram exhibited excellent predictive performance. The difference in predictive ability between the novel nomogram and the conventional FIGO stage system was further evaluated. The C-indexes of the proposed nomogram were higher than those of the FIGO staging system in both the training and validation cohorts, implying that the novel model had better discrimination ability than the conventional one. The DCA curves indicated that the nomogram model exhibited consistent advantages in clinical usefulness compared to the FIGO staging system. Furthermore, the NRI and IDI values demonstrated that the novel model had a better predictive ability than the conventional FIGO stage system.

Based on the total score of all predictors in the nomogram model, a novel risk classification system was established to stratify women with ASCC in the training cohort into three prognostic groups, including the low-, medium- and high-risk groups. Three significantly distinguishable survival curves revealed that the risk classification model effectively recognized high-risk ASCC patients, and this discrimination ability was further confirmed in the validation and total cohort. The constructed nomogram and the novel risk stratification system are convenient and easy-to-use scoring systems and might serve as useful tools for clinicians to more precisely estimate the prognosis of ASCC patients and stratify subgroups of patients who need a specific treatment strategy.

Currently, the SEER database comprises 21 cancer registries, covering more than one-third of the US population, and provides valid and reliable data. Clinical prediction models based on SEER data present quality assurance and persuasiveness (33). Although our study was carefully conducted on a large sample size of patients in the United States, several limitations still exist. First, data regarding the nutritional status, progression or recurrence, and second or later treatment course were not available. These factors might likely affect the critical aspects of disease progression in an oncological study. Besides, we were unable to record each variable for every patient. For example, the information regarding tumor size was missing for most patients, and thus, the inclusion or exclusion of patients with missing data might have affected the study results. Lastly, our study was retrospective in nature; thus, inherent selection biases were inevitable. Therefore, well-designed prospective studies are warranted to further verify the conclusions of our study.

## Conclusions

This study comprehensively analyzed the incidence, prognostic factors, and treatment outcomes of ASCC patients in a large population. The incidence of ASCC declined between 2000 and 2017. The proportion of young, married, high grade, early FIGO stage, small tumor size, and patients receiving more radical surgery in the ASCC group were significantly higher than those in the SCCC group. FIGO stage, surgical procedure, tumor size, age, and tumor grade were independent prognostic factors for CSS in ASCC patients. Adjuvant radiotherapy or chemotherapy was not recommended in patients with ASCC at an early stage. An accurate predictive nomogram was established and validated. A novel risk stratification system based on the established nomogram could effectively distinguish high-risk ASCC patients.

## Data Availability Statement

The original contributions presented in the study are included in the article/**Supplementary Material**. Further inquiries can be directed to the corresponding author.

## Author Contributions

PC, LY, CC, and XC analyzed the data and drafted the manuscript. ZL conceived the study and revised the manuscript. All authors contributed to the article and approved the submitted version.

## Funding

This study was supported by Grants from the Department of Science and Technology of Jilin Province (No. 3D5190668428) and the Jilin Province Development and Reform Commission (No. 3J117B963428).

## Conflict of Interest

The authors declare that the research was conducted in the absence of any commercial or financial relationships that could be construed as a potential conflict of interest.
