# Supplementary material for: Adenosquamous Carcinoma of the Cervix: A Population-Based Analysis
Source: Front Oncol. 2021 Jul 22;11:652850. doi: 10.3389/fonc.2021.652850 (PMC8339955; doi:10.3389/fonc.2021.652850)
Supplement: Supplementary file 1 [file DataSheet_1.docx]

**Adenosquamous carcinoma of the cervix: a population-based analysis**


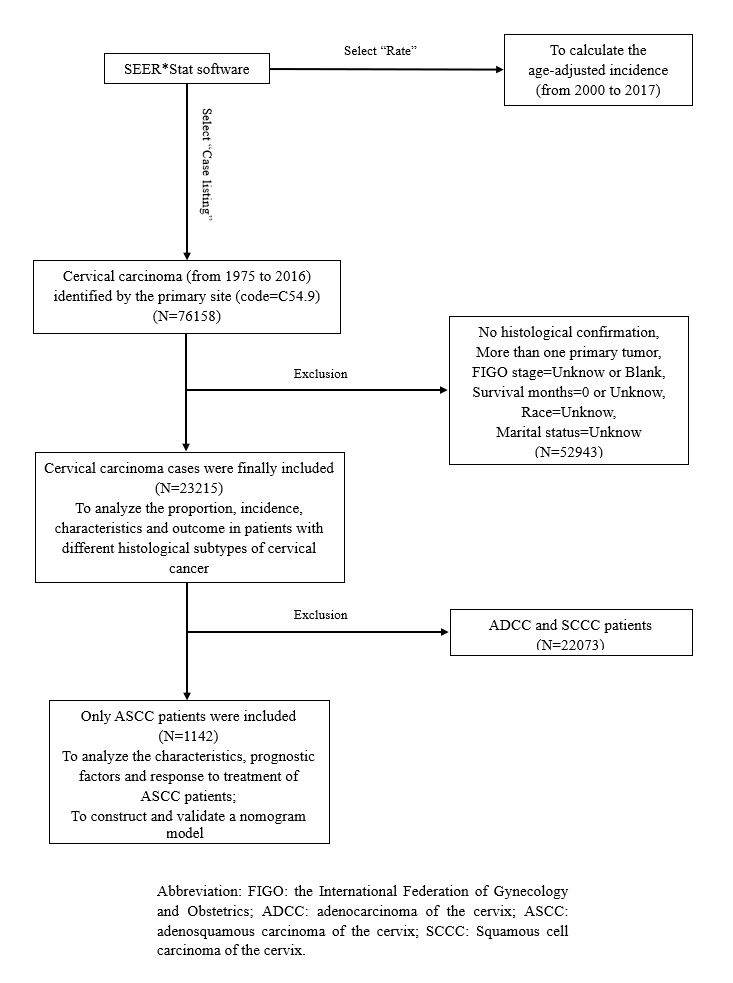


Figure S1. Schematic representation of patient selection and study design.

Table S1. Clinical characteristics of cervical cancer among different histological subtypes.

| **Variables** | **ADCC**  N=3012 | **ASCC**  N=1142 | **SCCC**  N=17339 | ***P* value** |
| --- | --- | --- | --- | --- |
| **Age (years)** |  |  |  | <0.001 |
| ≤60 | 2421 (80.4) | 962 (84.2) | 13495 (77.8) |  |
| >60 | 591 (19.6) | 180 (15.8) | 3844 (22.2) |  |
| **Marital status** |  |  |  | <0.001 |
| Married | 2013 (62.7) | 716 (62.7) | 9852 (56.8) |  |
| Unmarried | 776 (25.8) | 352 (30.8) | 5802 (33.5) |  |
| Widowed | 213 (7.1) | 74 (6.5) | 1685 (9.7) |  |
| **Race** |  |  |  | <0.001 |
| Black | 233 (7.7) | 132 (11.6) | 2752 (15.9) |  |
| White | 2431 (80.7) | 865 (75.7) | 12777 (73.7) |  |
| Other^*^ | 348 (11.6) | 145 (12.7) | 1810 (10.4) |  |
| **Grade**^#^ |  |  |  | <0.001 |
| I/II | 1497 (49.7) | 328 (28.7) | 6801 (39.2) |  |
| III/IV | 727 (24.1) | 588 (51.5) | 7074 (32.9) |  |
| Unknow | 788 (26.2) | 226 (19.8) | 5793 (27.0) |  |
| **FIGO stage** |  |  |  | <0.001 |
| I | 1806 (60.0) | 565 (49.5) | 7544 (43.5) |  |
| II | 336 (11.2) | 161 (14.1) | 2777 (16.0) |  |
| III | 463 (15.4) | 260 (22.8) | 4473 (25.8) |  |
| IV | 407 (13.5) | 156 (13.7) | 2545 (14.7) |  |
| **Tumor size (cm)** |  |  |  | <0.001 |
| ≤3.5 | 1183 (39.3) | 434 (38.0) | 4784 (27.6) |  |
| >3.5 | 840 (27.9) | 419 (36.7) | 6469 (37.3) |  |
| Unknow | 989 (32.8) | 289 (25.3) | 6086 (35.1) |  |
| **Surgical procedure** |  |  |  | <0.001 |
| None | 933 (31.0) | 348 (30.5) | 8474 (48.9) |  |
| LTD | 338 (11.2) | 106 (9.3) | 2167 (12.5) |  |
| THR-RTO | 210 (7.0) | 57 (5.0) | 1132 (6.5) |  |
| THR+RTO | 667 (22.1) | 255 (22.3) | 2612 (15.1) |  |
| RHR | 864 (28.7) | 376 (32.9) | 2954 (17.0) |  |
| **Radiotherapy** |  |  |  | <0.001 |
| No | 1695 (56.3) | 451 (39.5) | 6617 (38.2) |  |
| Yes | 1317 (43.7) | 691 (60.5) | 10722 (61.8) |  |
| **Chemotherapy** |  |  |  | <0.001 |
| No | 1829 (60.7) | 541 (47.4) | 8076 (46.6) |  |
| Yes | 1182 (39.3) | 601 (52.6) | 9263 (53.4) |  |

^*^ including Asian, American Indian and Alaska Native.

^#^ I: well differentiated; II: moderately differentiated; III: poorly differentiated; IV: undifferentiated.

Abbreviation: ADCC: adenocarcinoma of cervix; ASCC: adenosquamous carcinoma of cervix; SCCC: squamous cell carcinoma of cervix; FIGO: the International Federation of Gynecology and Obstetrics; LTD: local tumor destruction; THR-RTO: total hysterectomy without removal of tubes and ovaries; THR+RTO: total hysterectomy with removal of tubes and ovaries; RHR: radical hysterectomy.


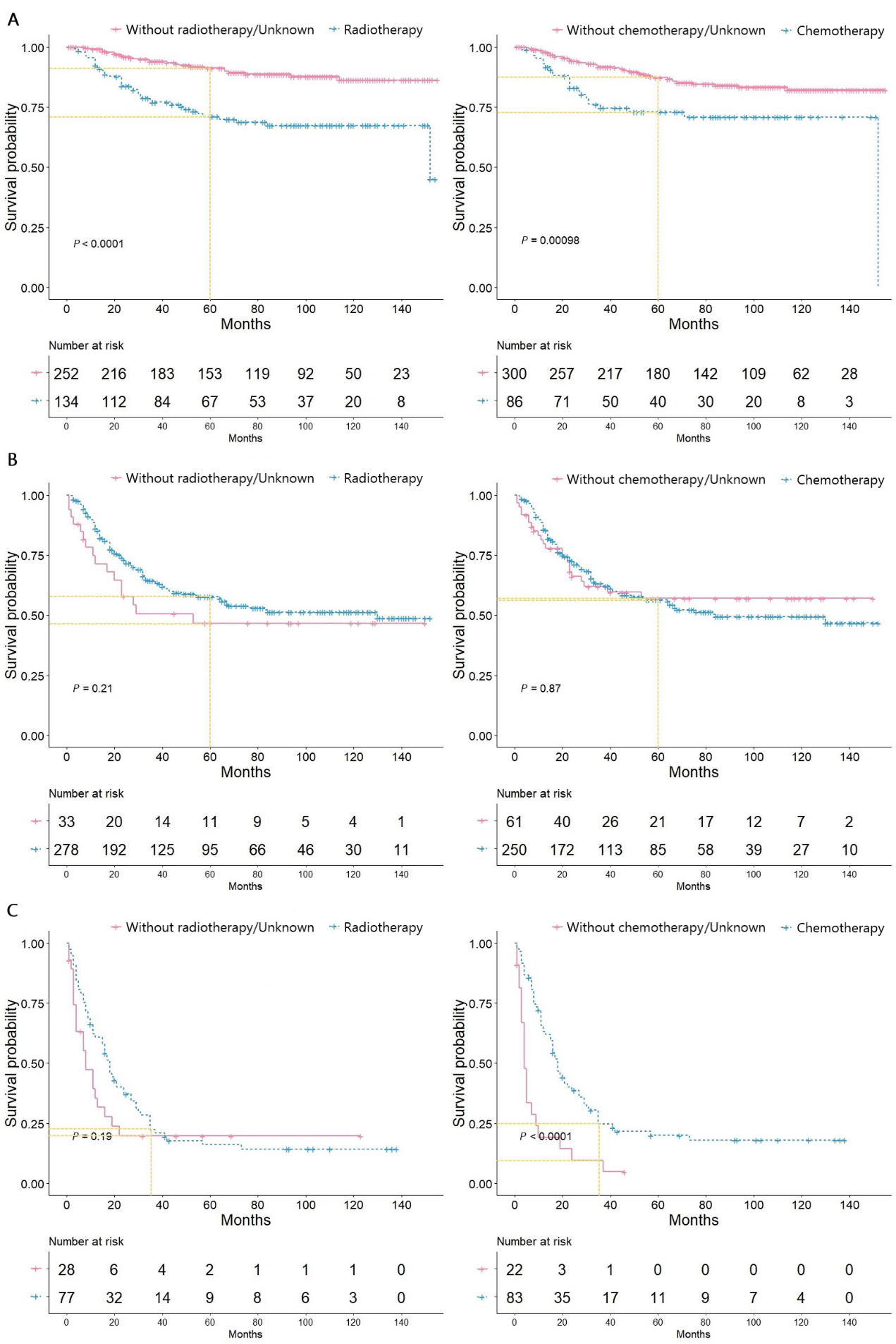


Figure S2. Effect of chemotherapy and radiotherapy on cancer-specific survival in ASCC patients at different stages. Local stage (A): radiotherapy vs. non-radiotherapy/unknown, chemotherapy vs. non-chemotherapy/unknown; regional stage (B): radiotherapy vs. non-radiotherapy/unknown, chemotherapy vs. non-chemotherapy/unknown; distant-stage (C): radiotherapy vs. non-radiotherapy/unknown, chemotherapy vs. non-chemotherapy/unknown.
